# Staged transthoracic approach to persistent atrial fibrillation (TOP-AF): study protocol for a randomized trial

**DOI:** 10.1186/1745-6215-15-190

**Published:** 2014-05-26

**Authors:** Claudio Pragliola, Pasquale Mastroroberto, Mario Gaudino, Massimo Chello, Elvio Covino

**Affiliations:** 1 Dipartimento di Scienze Cardiovascolari, Policlinico A Gemelli, Largo Gemelli 8, 00168 Roma, Italy; 2 Dipartimento di Scienze Mediche e Chirurgiche, Università della Magna Grecia, Viale Europa, Germaneto, 88100 Catanzaro, Italy; 3 UOC Cardiochirurgia, Università Campus Biomedico, Via A. Del Portillo 200, 00128 Roma, Italy; 4 Dipartimento di Scienze Cardiovascolari, Università Cattolica S. Cuore, Policlinico A Gemelli, Largo A Gemelli 8, 00168 Roma, Italy

**Keywords:** Atrial fibrillation, Persistent atrial fibrillation, Ablation, Surgical ablation

## Abstract

**Background:**

Persistent atrial fibrillation frequently shows multiple different electrophysiological mechanisms of induction. This heterogeneity causes a low success rate of single procedures of ablation and a high incidence of recurrence. Surgical ablation through bilateral thoracotomy demonstrates better results after a single procedure. Prospective observational studies in inhomogeneous populations without control groups report a remarkable 90% of success with hybrid or staged procedures of surgical ablation coupled with catheter ablation. In this trial, we will examine the hypothesis that a staged approach involving initial minimally invasive surgical ablation of persistent atrial fibrillation, followed by a second percutaneous procedure in case of recurrence, has a higher success rate than repeated percutaneous procedures.

**Methods/Design:**

This is a controlled (2:1) randomized trial comparing use of a percutaneous catheter with minimally invasive transthoracic surgical ablation of persistent atrial fibrillation. The inclusion and exclusion criteria, definitions, and treatment protocols are those reported by the 2012 Expert Consensus Statement on catheter and surgical ablation of atrial fibrillation. Patients will be randomized to either percutaneous catheter (*n* = 100) or surgical (*n* = 50) ablation as the first procedure. After 3 months, they are re-evaluated, according to the same guidelines, and receive a second procedure if necessary. Crossover will be allowed and data analyzed on an “intention-to-treat” basis. Primary outcomes are the incidence of sinus rhythm at 6 and 12 months and the proportions of patients requiring a second procedure.

**Discussion:**

The use of a staged strategy combining surgical and percutaneous approaches might be more favorable in treatment of persistent atrial fibrillation than the controversial single percutaneous ablation.

**Trial registration:**

ISRCTN08035058 Reg 06.20.2013

## Background

Persistent atrial fibrillation (PeAF) is defined as episodes of atrial fibrillation (AF) lasting more than 7 days or requiring an electric or pharmacologic cardioversion to restore the sinus rhythm (SR) [1]. PeAF is frequently caused by different coexistent electrophysiological mechanisms of induction [2]. This complex origin frequently requires the use of a combination of ablation techniques to achieve acceptable results. Indeed, pulmonary vein isolation (PVI) [3] and the ablation of complex fractioned atrial electrograms (CFAEs) [4] did not result in satisfactory results when used separately during percutaneous catheter ablation procedures (PCAs), whereas the rate of success increased to 74% after combining the two techniques and was 88% when ablation was repeated after 3 months [5]. The evaluation of treatment for PeAF is further complicated by the small number of patients with PeAf included in controlled trials [6]. Similar outcomes are observed for the surgical treatment of PeAF. For example, in the FAST trial (atrial fibrillation catheter ablation versus surgical ablation treatment) [7], surgical ablation achieved better results (65% versus 36%; *P* = 0.0022) than PCA in complex patients, whereas this trend was not confirmed in the cases with PeAf, who were a minority of those randomized.

Regardless of the mechanism involved, the majority of the sites of origin of PeAF are located in the posterior part of the left atrium, as this portion is easily encompassed by the box lesions created by surgical ablations [8]. As such, a staged approach may be a more suitable strategy, with use of percutaneous endocardial techniques only in case of failure of the surgical procedures. Recently, Pison [9] obtained a success rate of 90% in PeAf with a hybrid approach involving bilateral minimal surgical access and PCA. In this series, only 23% of the lines created surgically were not transmural, and 88% of the patients had the AF terminated by a posterior left atrial isolation. Comparable results were reported with staged [10] approaches of thoracoscopy or transabdominal pericardial [11] access and PCA.

In the present trial, we examine the hypothesis that a staged approach involving minimally invasive surgical ablation of persistent atrial fibrillation, followed by a second percutaneous procedure in cases of recurrence, will have a success rate at 12 months higher than repeated percutaneous procedures. According to the recently published European guidelines on surgical ablation of AF alone, we will select patients with a class IIb indication to surgery and use the class I bipolar radiofrequency as the energy source for the ablation [12].

## Methods

The overall hypothesis of this study is that an initial surgical procedure has a higher probability of success than an initial percutaneous ablation, and therefore, fewer patients will be submitted to a second ablation. The proposed therapies are currently accepted and considered Class II indications in the current European Society of Cardiology [13] and European Association of Cardiothoracic Surgery [12] guidelines for the treatment of PeAf. As such, their comparison complies with the Declaration of Helsinki for the Ethical Principles of Medical Research in human subjects. The Ethics Committee of the University of Magna Grecia in Catanzaro (Italy) gave full approval to the study without conditions. No external financial support exists. Full reimbursement of the cost of the therapies has already been granted by the Italian National Health System.

All participants will be required to sign an informed consent to adhere to the study. The Surgical Staged Approach (SSA) strategy consists of an initial minimally invasive surgical ablation, followed by a percutaneous catheter ablation if necessary. To avoid any possible bias introduced by the difference in surgical techniques, we will use only the bipolar radiofrequency using the Estech cobra fusion ablator (Estech is a Market brand of Atricure Inc West Chester 45069 Ohio USA) introduced through a right minimal thoracotomy. The control group will receive the current standard strategy of PCA. In the case of recurrence of the AF, the second procedure will be either percutaneous or surgical. The inclusion criteria and the protocols for managing the pharmacologic and anticoagulant therapy, the description of the surgical approach, and the technique of the percutaneous ablation are those indicated by the 2012 HRS/EHRA/ECAS expert consensus statement on catheter and surgical ablation of AF [12] and the European Guidelines for the treatment of lone AF [13].

### Inclusion criteria

• Patient age ≥18 years.

• Patients with persistent AF defined as at least one sustained episode lasting more than 7 days in the last 12 months.

• Patients with symptomatic AF that is refractory to at least one antiarrhythmic medication; symptomatic patients are those who have been aware of their AF at any time within the last 5 years before enrolment. Symptoms may include, but are not restricted to, palpitations, shortness of breath, chest pain, fatigue, left ventricular dysfunction, or other symptoms, or any combination of these.

• At least one episode of persistent AF must have been documented by ECG, Holter, loop recorder, telemetry, transtelephonic monitor, or implantable device within last 2 years of enrollment in this investigation.

• Patients must be able and willing to provide written informed consent to participate in this investigation.

• Patients must be willing and able to comply with all periablation and follow-up requirements.

### Exclusion criteria

• Patients with paroxysmal AF, defined as a sustained episode lasting <7 days.

• Patients with long-standing persistent AF lasting >1 year.

• Patients for whom cardioversion or sinus rhythm will never be attempted/pursued.

• Patients with AF secondary to a reversible cause.

• Patients with contraindications to systemic anticoagulation.

• Patients with left atrial size ≥55 mm (2-dimensional echocardiography, parasternal long-axis view).

• Patients with LA thrombi as demonstrated by transesophageal echocardiography (TEE).

### Interventions

#### *Antiarrhythmic therapy*

All antiarrhythmic drugs will be discontinued 5 half-lives before the procedure. In exceptional cases requiring strict rhythm management to control preoperative symptoms, this will be managed with intravenous esmolol. Amiodarone requires at least 8 weeks of suspension.

#### *Anticoagulant therapy*

Patients receiving warfarin will stop the medication and switch to LMW heparin, which will be maintained during the procedure but which can be controlled in case of major bleeding. The same applies to patients receiving apixaban, dabigatran, or rivaroxaban, to control the silent thromboembolism described during the creation of endocardial lesions [14]. If the patient is taking only aspirin, it need not be discontinued.

All patients will receive preoperative TEE echocardiograms to rule out the presence of left atrial thrombi and to determine the size, morphology, and flow pattern in the left atrial appendage.

### Staged surgical ablation group

Fifty patients will be randomized to surgical ablation of AF. The ablation procedure will be performed with the use of Estech cobra fusion, according to the manufacturer’s instructions and to the commonly described techniques [10,12,15]. Up to three applications will be allowed to ablate the left atrial box. Bidirectional block will be checked, and reasons for not obtaining it will be noted. Inducibility of AF at the end of the procedure will be recorded. Cardioversion will be allowed to restore SR. At the end of the procedure, the patient will be awakened and transferred to the ward. An interval of 3 months will be allowed after the initial ablation procedure, as per the Heart and Rhythm European Society Association/European Heart Rhythm Association/European Cardiac Arrhythmia Society expert consensus statement [13]. During this period, the recurrences of AF, atrial tachycardias, and atrial flutter will not be counted toward the primary or secondary end points. Antiarrhythmic medications may be continued for the first 3 months to avoid early recurrences. At 3 months, they must be stopped to assess recurrences. For patients continuing to have recurrence controlled by the therapy, it will be left to the preference of the investigator to continue the medical therapy or to refer the patient for a PCA.

### Percutaneous catheter ablation group

One hundred patients will be randomized to PCA and treated according to the protocol of the referring center. In all cases, complete PVI must be at least attempted and confirmed by bidirectional block. The referring investigator can then add any of the current methods of PeAF treatment, including substrate modification with the infusion of isoproterenol to uncover non-PVI triggers and CFAE. Lines can be drawn at the discretion of the investigators. Inducibility of AF at the end of the procedure will be recorded. Cardioversion will be allowed to restore SR.

After the 3-month blanking period, it will be left to the investigator’s preference to refer the patients with PeAF recurrence to a second PCA procedure, surgical ablation, or switch to a rate-control approach. The latter will be counted as a failed repeated procedure.

### Assessment of recurrence

The recurrence will be assessed by clinical follow-up at 1, 3, 6, 9, and 12 months with 12-lead ECG + 24 Holter monitoring. Transthoracic echocardiography will be also performed to record the presence of an atrial contraction in case of sinus rhythm. Quality of life will be assessed by a modified 36-item Short Form Health Survey and the Euro 5D questionnaire. The primary outcomes include the freedom from AF at 12 months, as detected by a 12-lead ECG and a 24-hour Holter examination after 3, 6, and 9 months of follow-up, and the occurrence of a second ablation procedure. The secondary outcomes include the duration of the procedures, freedom from any documented atrial arrhythmia, incidence of procedural complications, any late complication related to the procedures, and quality of life.

### Statistical analysis

**Figure 1:**
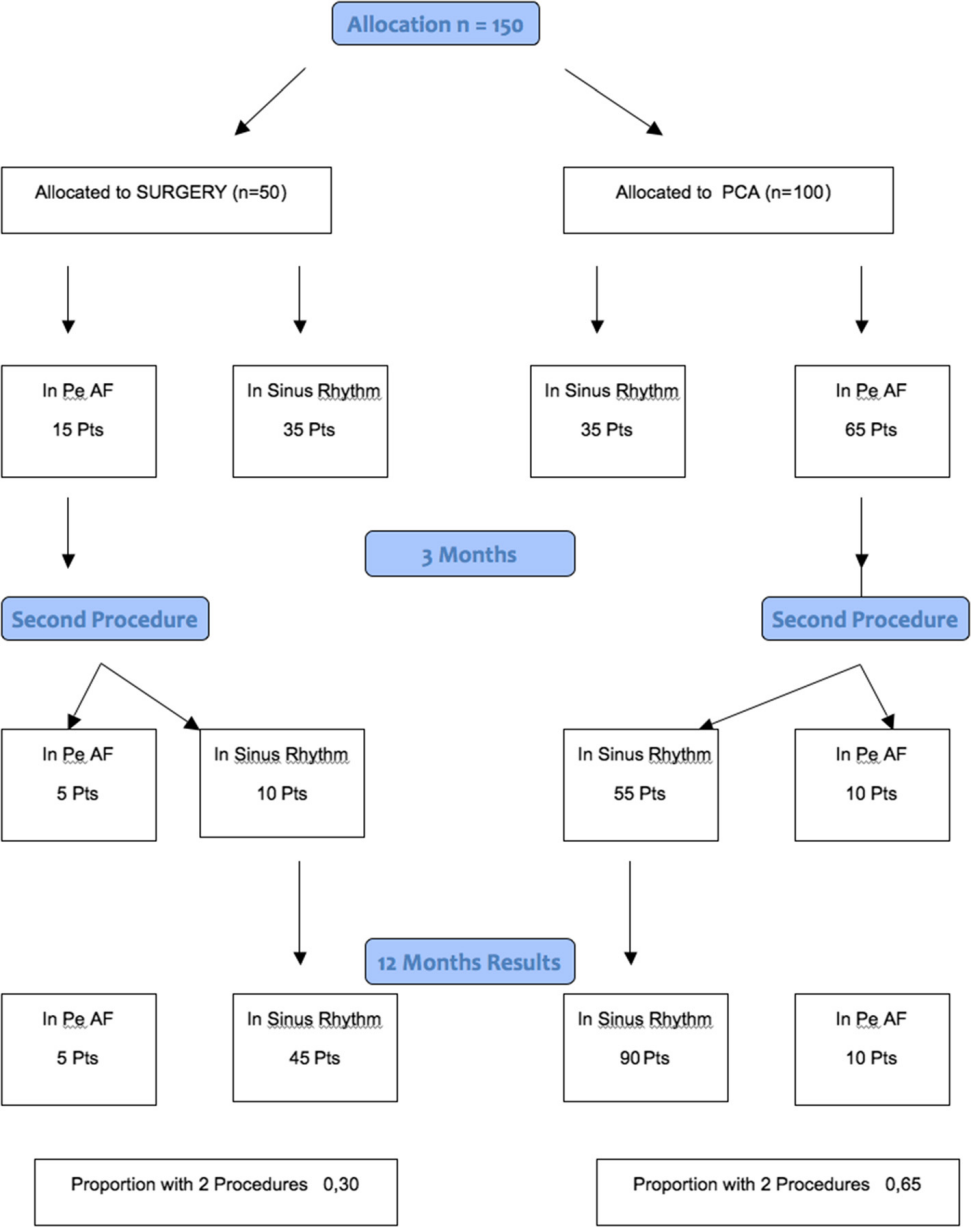
Flow chart of the study.

Randomization will be performed by using currently available computer software. Sample size is based on the hypothesis that the success rate of the first procedure will be approximately 70% for the surgical cases and 35% to 40% for the PCA cases. At the end of the interventions, the number of patients in SR should be equivalent in the two groups, whereas the proportions of those submitted to a second procedure, either percutaneous or surgical, should differ significantly. Variables will be analyzed with an intention-to-treat method. The three possible results of this study are described in Table 1. A flow chart depicting the most favorable result in which the power of the test is 98% is shown in Figure 1. Data will be expressed as mean ± SD and 95% confidence limits, as appropriate. Comparisons between patient groups will be performed with the Mann–Whitney test for continuous variables and the χ^2^ test for categoric variables. The *z* test and the odds ratio will be used for differences between proportions of patients receiving repeated procedures in the two groups. A value of *P* < 0.05 will be considered statistically significant. Time-to-event distributions will be estimated by the Kaplan-Meier method and compared with the log-rank test. The Cox proportional hazards model will be used to identify independent risk factors for recurrences. Stepwise selection procedure will be adopted. A value of *P* < 0.05 will be considered significant for variable entry for stepwise selection. Analyses are performed with the SPSS 18.0 software package (SPSS, Inc, Chicago, IL, USA).

**Table 1:** Anticipated results for different success rates of first PCA procedure

**% of SR in the PCA group after first procedure**	**35%**	**45%**	**50%**
**χ**^**2 **^**test for first procedure**	*P* < 0.0001	*P* = 0.007	*P* = 0.03
Power of the test	99%	84%	65%
**Strength of association**			
Relative risk	2.2	1.8	1.66
95% confidence interval	1.4 to 3.4	1.2 to 2.9	1.0 to 2.6
Odds ratio	4.3	2.9	2.33
95% confidence interval	2.1 to 9.0	1.4 to 5.9	1.1 to 4.7
**Difference between proportions**			
Fraction of second procedures in the two groups	0.65-0.30	0.55-0.30	0.50-0.30
Difference between fractions	0.35	0.25	0.2
95% confidence interval of difference	0.1 to 0.5	0.1 to 0.4	0.0 to 0.3

## Discussion

PeAf is more complex to treat than the simple paroxysmal AF. Several different percutaneous and surgical techniques have been proposed for its treatment, owing to the difficulties in the standardization of the ablation procedures. In addition, many reports dealing with the ablation of PeAF also include patients affected by paroxysmal and permanent fibrillation, which introduces a considerable selection bias.

As in any arrhythmia, a number of basic problems are involved in ablating the PeAF, including the following: (a) identifying the mechanism; (b) locating the source; (c) reaching the ablation area; (d) choosing the lesion set; and (e) consistently obtaining transmural lesions. As PeAf shows heterogeneity in its mechanism of origin, a simple PVI has unpredictable results. Thus, other techniques such as CFAE ablation and linear ablations, with or without substrate control, have been developed to increase the percentages of termination of the arrhythmia. Once the arrhythmia has been ablated, it can also recur in other parts of the atrium with a different mechanism, causing a high percentage of recurrent fibrillation. Recently, focal ablation and the rotor modulation technique (FIRM) [16] have shown promising results in a limited number of cases. Surgical ablations that de-connect a large part of the posterior left atrium, where many of the sources of the PeAF are located, have also become increasing used owing to their simple approach. Conversely, surgical procedures also are difficult to evaluate, and numerous observational studies of mixed populations without controls used different sources of energy and line combinations [15,17,18].

The FAST trial [7] was the first to compare the surgical and the percutaneous approaches, in which patients randomized to surgery had a higher rate of termination of the arrhythmia. However, their results were compromised by the incidence of complications. Many of these complications (pneumothorax, rib fracture) could be easily avoided by careful analysis of the results and by experience, which eventually results in an equivalent rate of complications between the two experiment groups. Further, the surgical ablation in that study was performed through a bilateral minimal thoracotomy. This approach was necessary to accomplish a complete surgical PVI with the use of bipolar radiofrequency delivered by the Atricure device. The bilateral approach adds time and increases the chances of complications. This can be avoided with different devices such as the Estech Cobra Fusion, which is capable of a complete electrical deconnection of the posterior left atrium, delivering bipolar radiofrequency through a single right minimally invasive approach. According to the European Guidelines [12], bipolar radiofrequency is the preferred source of energy for achieving a complete transmural line of ablation [19]. The transmurality of the lesions can be consistently obtained with hybrid or simultaneous [9] and staged or sequential [10] approaches.

The hybrid simultaneous procedures have some disadvantages, in that they take time and do not allow a blank period to evaluate the clinical efficacy of surgery. The staged procedures allow a blank period of evaluation, and two modalities of ablation (surgical and percutaneous) may be used after a predetermined period or only in case of recurrences.

All of these procedures share the same basic principles. The posterior left atrium (the portion where many of the sources of PeAf are located) must be electrically separated from the base of the heart [8], with the addition of some endocardial lines, if necessary. In our study, we have chosen to adopt a staged approach. This has some possible advantages, including the evaluation of the efficacy of a monolateral minimally invasive approach by using bipolar radiofrequency. For this, we will use the Estech Cobra Fusion system. The ablation device encircles the posterior portion of the left atrium and allows the use of bipolar radiofrequency in a controlled protocol. Second, the procedural time is expected to be relatively short so as to avoid any ICU stay. Third, a blank period will allow a second percutaneous ablation to be performed only in patients with recurrent AF, which should reduce the total number of invasive procedures necessary to control the AF.

Finally, in our opinion, this will allow us to study the modalities of failures of the surgical approach and give valuable information in refinement of the techniques and devices.

### Study limitations

No standard strategy is known for the percutaneous ablation of PeAF. In the PCA control group of our study, the choice of the initial technique will be left to the treating cardiologist (PVI, CFAE ablation with or without manipulation of the substrate, or rotor manipulation are frequently used in combination or in staged procedures). Insulated PVI is the least effective technique, whereas rotor modulation has been recently introduced and is not widely used. Thus, we expect that the majority of the patients in the control group will undergo a combination of PVI and CFAE ablation. In cases of failure in the surgical group, this choice will be dictated by the residual electrophysiological findings. This is a potential limitation of the study that may make comparison between the two groups difficult.

## Trial status

At the moment of submission, the trial is recruiting patients.

## Abbreviations

AF: Atrial fibrillation; CFAE: complex fractioned atrial electrogram; ECAS: European Cardiac Arrhythmias Society; ECG: electrocardiogram; EHRA: European Hearth Rhythm Association; FAST: atrial fibrillation catheter ablation versus surgical ablation treatment; HRS: Heart Rhythm Society; LMW: low molecular weight; PCA: percutaneous catheter ablation; PeAF: persistent atrial fibrillation; PVI: pulmonary vein insulation; SR: sinus rhythm; TEE: transesophageal echo; PAF: paroxysmal atrial fibrillation; SSA: staged surgical ablation.

## Competing interests

The authors do not have any financial relationship, nor have they received financial, property, or intellectual aid from any commercial source.

## Authors’ contributions

Each author substantially contributed to the study and met the authorship criteria. CP, conception, design, manuscript writing, and revision. PM, design and statistical analysis. MG, study design and critical revision. MC, statistical analysis, manuscript writing, and critical revision. EC, conception, critical revision, and manuscript revision. All the authors read and approved the final manuscript.
